# Quantifying the pediatric surgical need in Uganda: results of a nationwide cross-sectional, household survey

**DOI:** 10.1007/s00383-016-3957-3

**Published:** 2016-09-10

**Authors:** Elissa K. Butler, Tu M. Tran, Anthony T. Fuller, Alexa Brammell, Joao Ricardo Vissoci, Luciano de Andrade, Fredrick Makumbi, Samuel Luboga, Christine Muhumuza, Vincent F. Ssennono, Jeffrey G. Chipman, Moses Galukande, Michael M. Haglund, Emily R. Smith

**Affiliations:** 1 Department of Surgery, University of Washington, Seattle, WA USA; 2 Duke University Global Health Institute, 310 Trent Drive, Durham, NC 27710 USA; 3 Division of Global Neurosurgery and Neuroscience, Duke University, Durham, NC USA; 4 Duke University School of Medicine, Durham, NC USA; 5 State University of West of Parana, Unioeste, Foz do Iguaçu, Brazil; 6 Public Health Research Group, Unioeste, Toledo, Brazil; 7 Makerere University School of Public Health, Kampala, Uganda; 8 Department of Anatomy, Makerere University School of Medicine, Kampala, Uganda; 9 Ministry of Health, Government of Uganda, Kampala, Uganda; 10 Department of Surgery, University of Minnesota, Minneapolis, MN USA; 11 Department of Surgery, Makerere University College of Health Sciences, Kampala, Uganda; 12 Department of Neurosurgery, Duke University Medical Center, Durham, NC USA

**Keywords:** Global surgery, Community survey, Low- and middle-income countries, Sub-Saharan Africa, SOSAS

## Abstract

**Purpose:**

Little is known about the prevalence of pediatric surgical conditions in low- and middle-income countries. Many children never seek medical care, thus the true prevalence of surgical conditions in children in Uganda is unknown. The objective of this study was to determine the prevalence of surgical conditions in children in Uganda.

**Methods:**

Using the Surgeons OverSeas Assessment of Surgical Need (SOSAS) survey, we enumerated 4248 individuals in 2315 households in 105 randomly selected clusters throughout Uganda. Children aged 0–18 were included if randomly selected from the household; for those who could not answer for themselves, parents served as surrogates.

**Results:**

Of 2176 children surveyed, 160 (7.4 %) reported a currently untreated surgical condition. Lifetime prevalence of surgical conditions was 14.0 % (305/2176). The predominant cause of surgical conditions was trauma (48.4 %), followed by wounds (19.7 %), acquired deformities (16.2 %), and burns (12.5 %). Of 90 pediatric household deaths, 31.1 % were associated with a surgically treatable proximate cause of death (28/90 deaths).

**Conclusion:**

Although some trauma-related surgical burden among children can be adequately addressed at district hospitals, the need for diagnostics, human resources, and curative services for more severe trauma cases, congenital deformities, and masses outweighs the current capacity of hospitals and trained pediatric surgeons in Uganda.

## Introduction

With the highest estimated need for surgery worldwide and the most limited access to healthcare resources [1], countries in sub-Saharan Africa experience a disproportionate unmet need for surgical care. The Disease Control Priorities Project estimated that at least 11 % of the total global burden of disease is attributable to surgically treatable diseases [2], which is likely an underestimate, given recent population-based surgical assessments [3–5]. Among children, the burden of surgical conditions is even more striking; approximately 85 % of children in Africa have a surgically treatable condition by the age of 15 [6]. Many pediatric surgical conditions are congenital, and, thus, carry the risk of life-long disability, disproportionately increasing the number of disability-adjusted life years (DALYs) lost [7].

Like many other countries in sub-Saharan Africa, surgical care is a low-to-moderate health priority in Uganda [8]. There are approximately 0.67 physicians per 100,000 people and 0.1 surgeons per 100,000 people [9], compared with 250 surgeons per 100,000 in the United States [10]. Moreover, there are only three practicing certified pediatric surgeons and one pediatric anesthetist in the country. Two of the surgeons practice at the national referral hospital in Kampala and one practices at the Eastern regional referral hospital. Under the auspices of the College of Surgeons of East, Central and Southern Africa (COSECSA), Uganda is able to train one pediatric surgeon per year [11].

To enhance the priority of surgical care among the national health agendas of low- and middle-income countries (LMICs), it is important to quantify the current burden of untreated surgical disease among children. A hospital-based survey in Southwestern Uganda estimated that 180 operations per 100,000 populations annually were completed for children under the age of 14, compared with 5892 per 100,000 in the United Kingdom. Mission or non-governmental organization hospitals performed 55 % of pediatric operations [12]. This study is limited by its regional specificity and the use of hospital-based data. In a study at the national referral hospital in Kampala, of 1338 pediatric surgical admissions over a 28 month period, 679 were congenital anomalies (50.3 %), 281 infections (28.1 %), and 130 malignancies (9.7 %) [13]. Many children never seek medical care, thus the true prevalence of surgical conditions in children in Uganda is underrepresented in these studies. The Surgeons OverSeas Asssessment of Surgical Need (SOSAS) tool [14] was developed to overcome these challenges through a cross-sectional, population-based, household survey which provides a national estimate of the surgical burden of disease.

The objective of this study was to determine the pediatric burden of surgical disease in Uganda, both treated and untreated, to inform future efforts in improving pediatric surgical care.

## Materials and methods

### Setting

Over 50 % of Uganda’s 34.9 million inhabitants are under 18 years of age. The country has the third highest birth rate in the world at 44 births per 1000 population [15]. Only 8.0 % of the nation’s GDP is spent on health compared with 17.9 % in the United States and 9.4 % in the United Kingdom [16]. Uganda is organized into 11 sub-regions and the capital city of Kampala. The majority of health care is delivered at a district hospital level, which is able to perform basic emergency surgical procedures, including Cesarean sections and hernia repairs. There are 15 referral hospitals, which deliver a higher level of care and two national referral hospitals, one which can deliver specialized surgical care, while the second one focuses primarily on psychiatric disorders [17]. A few mission and non-governmental organization hospitals also provide elective specialty surgical care.

### Sampling, survey, and data collection

Using the SOSAS survey, we completed a two-stage, cluster-based nationwide, household survey, as previously described [18]. In short, the Uganda Bureau of Statistics assisted in randomly selecting 105 enumeration areas (EA), clustered by geographic sub-region. In each EA, 24 households were randomly selected from a list of all households within the EA. In each household, two individuals were randomly selected to complete the SOSAS survey, independent of age or gender. The SOSAS survey contains: (1) a household component gathering household demographics and information on any deaths that occurred in the household in the previous year and (2) an individual component querying for surgical conditions occurring in each anatomic location. The SOSAS Uganda team used the Performance Monitoring and Evaluation 2020 (PMA2020) Uganda team for data collection. PMA2020 is a survey team with trained enumerators, already living in each EA who speak each local language, as well as English. The SOSAS team trained PMA2020 enumerators on the SOSAS survey and how to identify surgical conditions.

### Ethical considerations

Makerere University School of Medicine Research and Ethics Committee, Duke University Health System Institutional Review Board, and University of Minnesota Institutional Review Board approved this study prior to implementation. Enumerators obtained informed consent from each head of household and each individual participating in the survey, prior to initiation of the survey. For children less than 18, a parent or guardian provided informed consent, and children 8–18 provided assent. When necessary, parents or guardians assisted children in answering survey questions.

### Statistical analysis

Children were defined as survey respondents between the ages of 0 and 18 years. Because our enumerators were not medically trained, six blinded surgical trainees and surgeons assessed each documented condition and assigned a rating on a defined four-point scale [0-definitely not surgical, 1-likely not surgical, 2-likely surgical, and 3-definitely surgical (received surgery)]. For discordant coding, the case was discussed, and a final score was assigned based on consensus. Cases with a score of 2 or 3 were included as surgically treatable conditions. In addition, each case was coded as treated or untreated based on whether the patient received appropriate surgical care. The lifetime prevalence of pediatric surgical conditions included all children with a surgically treatable condition and the unmet pediatric surgical need included those with an untreated surgically treatable condition. We analyzed the data using SAS 9.4 (SAS Institute Inc, Cary, NC, USA) and stored the data in spreadsheets on Microsoft Excel 2010 (Microsoft Corp, Redmond, WA, USA). Household and individual cases were weighted using design weights for each EA, household-level and individual-level response rates, and known population counts of gender and age groupings from the Uganda Census 2014 data. Demographic data and mortality estimates were compared between children who reported a surgical condition and those that did not report a surgical condition and between children with treated surgical conditions and those with untreated surgical conditions, using a weighted model. Childhood prevalence of surgical conditions was mapped by district, stratified by age group, using QGIS version 2.8 [19]. Clinical characteristics among children who reported a surgical condition were stratified and compared by age group and treatment status, using a weighted model. Proportions and frequencies described pediatric household deaths by age group, anatomic location, and the type of condition.

## Results

### Demographic characteristics of children interviewed in SOSAS (*n* = 2176)

SOSAS Uganda interviewed a random sample of 4248 individuals (97.1 % response rate) in 2315 households (96.4 % response rate) with a total of 11,148 household members. Of the 4428 respondents, 2176 (49.1 %) were 18 years of age or less (Table 1). Of pediatric respondents, 305 (14.0 %) reported having a surgical condition at some point in their life, of which 51.1 % were untreated (156; 7.2 % of total). The median age of the children interviewed was 6.1 (IQR 2.5, 11.4) years, and the 0–5 year age group was the most prevalent. Among children interviewed, about half (50.2 %) were female and the majority (81.8 %) lived in a rural area. Most children reported being healthy in the 12 months prior to the interview; however, those with surgical conditions were more likely to report poor health compared with those without surgical conditions (25.3 versus 9.2 %, respectively; *p* < 0.0001). Children with surgical conditions made more visits to health facilities in the 12 months prior to the interview than children without surgical conditions (*p* = 0.0005). No differences were noted between children with surgical conditions and those without surgical conditions in regard to transportation time, wait time, and transportation costs to reach a primary, secondary, or tertiary care facility. Of children with surgical conditions, children with untreated conditions trended towards being younger and living in an urban area (*p* = 0.14 and *p* = 0.06, respectively). Children with untreated surgical conditions reported being less healthy (*p* < 0.0001) and making more frequent visits to health facilities (*p* < 0.0001). They were also more likely to have longer transportation times to primary care facilities (17.9 versus 9.5 min, *p* = 0.01). There was no difference in cost of transport to care facilities when comparing treatment status.

**Table 1:** Demographic characteristics of children interviewed in SOSAS, stratified by the presence of surgical condition and treatment status

	All children (*n* = 2176)	No surgical condition (*n* = 1871)	Surgical conditions (*n* = 305)	p*	Treated surgical conditions (*n* = 149)	Untreated surgical conditions (*n* = 156)	p**
	% (*n*)	% (*n*)	% (*n*)		% (*n*)	% (*n*)	
Age (years)							
0–5	43.9 (808)	48.3 (713)	35.0 (95)	0.001	28.9 (43)	33.3 (52)	0.14
6–9	20.1 (472)	20.6 (416)	17.1 (56)		17.4 (26)	19.2 (30)	
10–14	23.3 (531)	22.3 (445)	29.5 (86)		27.5 (41)	28.8 (45)	
15–18	12.7 (365)	11.8 (297)	18.3 (68)		26.2 (39)	18.6 (29)	
Gender							
Male	49.7 (1083)	49.7 (932)	51.3 (151)	0.61	50.3 (75)	48.7 (76)	0.48
Female	50.2 (1093)	50.3 (939)	48.7 (154)		49.7 (74)	51.3 (80)	
Village type							
Rural	81.8 (1780)	81.5 (1530)	81.9 (250)	0.90	84.6 (126)	79.5 (124)	0.06
Urban	18.2 (396)	18.5 (341)	18.1 (55)		15.4 (23)	20.5 (32)	
Geographic region							
Central	24.9 (496)	24.3 (414)	26.9 (82)	0.02	30.2 (45)	23.7 (37)	0.14
Eastern	29.5 (564)	31.3 (514)	16.4 (50)		16.8 (25)	16.0 (25)	
Western	23.3 (494)	22.2 (407)	28.5 (87)		24.8 (37)	31.4 (49)	
Northern	22.3 (622)	22.1 (536)	28.2 (86)		28.2 (42)	28.8 (45)	
Healthy in past 12 months							
Yes	88.6 (1933)	1701 (90.8)	232 (74.7)	0.0001	87.2 (130)	65.4 (102)	<0.0001
No	11.4 (243)	170 (9.2)	73 (25.3)		12.8 (19)	34.6 (54)	
Number of facility visits in past 12 months							
0	91.9 (2006)	93.7 (1756)	81.2 (250)	0.0005	88.6 (132)	75.6 (118)	<0.0001
1–3	5.9 (122)	4.5 (81)	14.2 (41)		9.4 (14)	17.3 (27)	
4–6	1.6 (33)	1.2 (21)	4.2 (12)		2.0 (3)	5.8 (9)	
>7	0.6 (15)	0.6 (13)	0.4 (2)		0 (0)	1.3 (2)	
Transport time, min (mean, SD)							
Primary care	14.8 (1.8)	15.0 (1.9)	13.8 (2.2)	0.62	9.5 (1.9)	17.9 (3.4)	0.01
Secondary care	34.0 (3.1)	34.6 (3.3)	30.7 (3.2)	0.31	26.7 (3.6)	34.6 (4.0)	0.09
Tertiary care	52.4 (4.3)	53.4 (4.6)	46.4 (5.2)	0.16	40.7 (5.8)	51.8 (6.5)	0.12
Wait time, min (mean, SD)							
Primary care	59.7 (3.9)	59.7 (3.9)	53.9 (6.2)	0.21	49.5 (4.5)	58.2 (9.8)	0.35
Secondary care	93.9 (6.9)	95.3 (6.9)	85.4 (9.7)	0.17	73.8 (9.5)	96.4 (13.8)	0.11
Tertiary care	150.0 (11.5)	152.8 (11.7)	135.1 (16.3)	0.20	112.9 (13.0)	156.1 (24.5)	0.07
Costs to facility							
Primary care							
<5000	82.9 (1801)	83.4 (1556)	80.5 (245)	0.85	81.9 (121)	79.0 (124)	–
5000–10,000	5.3 (117)	5.1 (95)	6.6 (22)		5.7 (10)	7.5 (12)	
>10,000	1.0 (21)	0.9 (18)	0.9 (3)		0.0 (0)	1.8 (3)	
Missing	10.8 (237)	10.7 (202)	11.9 (35)		12.4 (18)	11.7 (17)	
Secondary care							
<5000	58.3 (1235)	58.2 (1061)	59.1 (174)	0.15	64.5 (93)	54.1 (81)	0.12
5000–10,000	19.3 (413)	20.0 (368)	15.0 (45)		12.9 (20)	16.9 (25)	
>10,000	16.1 (392)	15.6 (323)	19.7 (69)		15.0 (26)	24.0 (43)	
Missing	6.2 (136)	6.2 (119)	6.2 (17)		7.5 (10)	16.9 (7)	
Tertiary care							
<5000	21.7 (500)	20.9 (413)	26.4 (87)	0.12	31.7 (52)	21.5 (35)	0.09
5000–10,000	22.9 (426)	22.4 (355)	26.7 (71)		27.1 (37)	26.3 (34)	
>10,000	51.8 (1180)	52.9 (1039)	44.9 (141)		39.7 (58)	49.9 (83)	
Missing	3.5 (70)	3.8 (64)	1.9 (6)		1.4 (2)	2.3 (4)	

* *p* value comparing children with surgical conditions to children without surgical conditions

** *p* value comparing children with untreated surgical conditions to children with treated surgical conditions

**Fig. 1:**
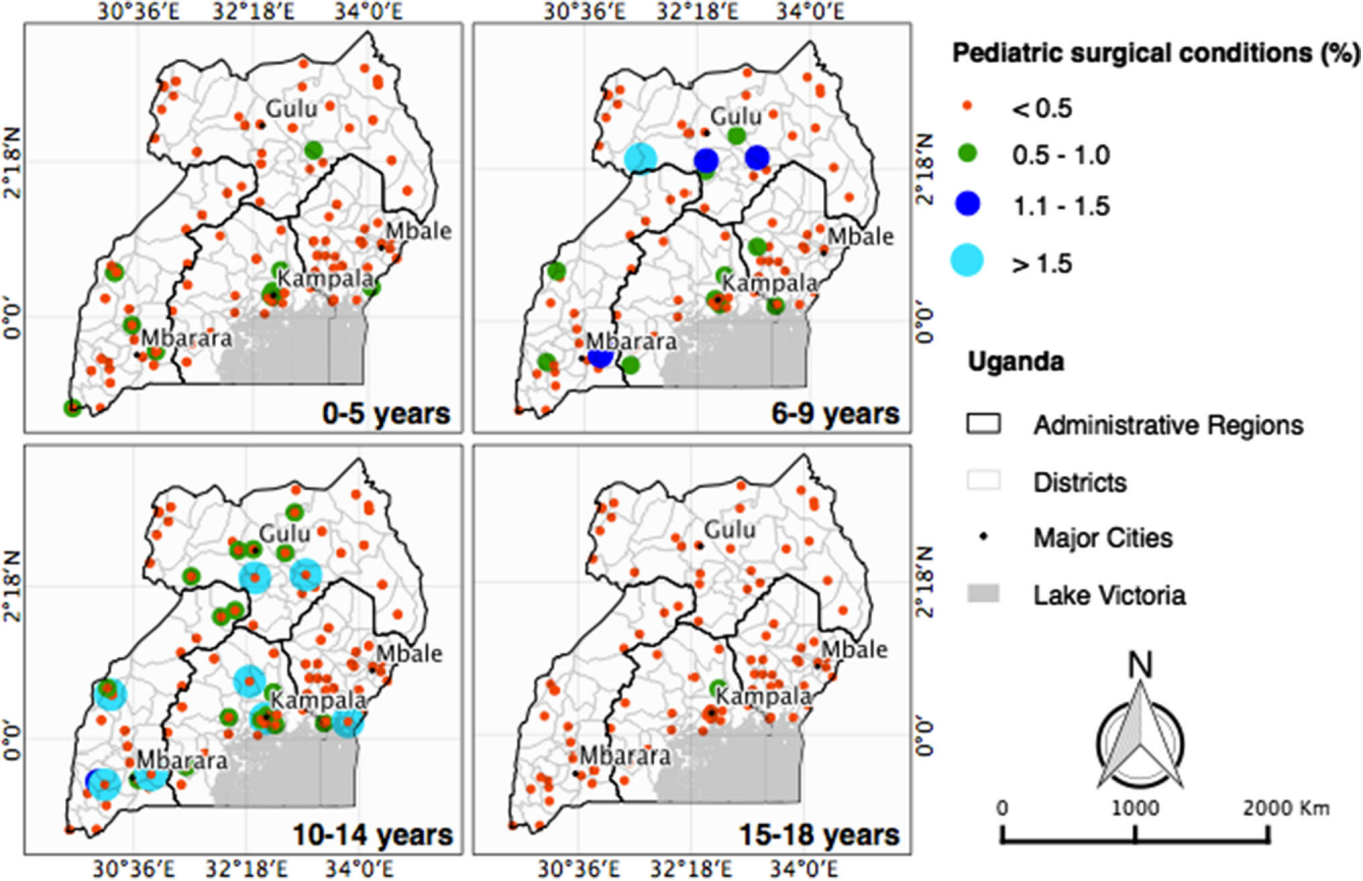
Geographic prevalence of pediatric surgical conditions in Uganda, by age. Each *dot* represents the proportion of pediatric surgical conditions, relative to the number of children within that region, by age group

Overall, pediatric surgical conditions had similar prevalence among the Central, Northern, and Western regions, but were less prevalent in the Eastern region. The geographic distribution of pediatric surgical conditions varied by age group (Fig. 1). Most of the surgical conditions among children less than 5 years were concentrated in the Central and Western regions of Uganda, while surgical conditions among 6 to 9 years old were more prevalent in the Western and Northern regions. Surgical conditions among older children were more evenly dispersed throughout Uganda. Although not statistically significant, children with treated surgical conditions trended towards residing in the Central region, compared to those with untreated surgical conditions, who were more likely to live in the Northern region (*p* = 0.14).

### Characteristics of children with surgical conditions, by age group (*n* = 305)

A total of 305 children reported 356 surgical conditions (Table 2). Overall, the most common anatomical areas were the face and neck (36.2 %) and the extremities and back (31.7 %). Younger children (less than 9 years) trended toward more face and neck conditions, and older children (greater than 9 years) had more extremity and back conditions (*p* = 0.066). The majority of children older than 5 years of age had been living with their condition for over a year. Masses (23.8 %), wounds due to injury (19.7 %), and acquired deformities (16.6 %) were the most common type of surgical conditions. The type of surgical condition varied by age group. Among children less than 6 years of age, masses and burns were most common, but among children older than 6 years, wounds, masses, and acquired deformities were more common (*p* = 0.058).

**Table 2:** Clinical characteristics and healthcare seeking for children with surgical conditions, by age

	Total % (*n*)	0–5 % (*n*)	6–9 % (*n*)	10–14 % (*n*)	15–18 % (*n*)	*p*
Number of individuals	305	35.0 (95)	17.1 (56)	29.5 (86)	18.3 (68)	
Number of conditions	356	31.5 (112)	17.1 (61)	27.8 (99)	23.6 (84)	
*Characteristics of condition*						
Location						
Face and neck	36.2 (129)	28.5 (34)	48.9 (30)	33.1 (37)	29.6 (28)	0.066
Extremity and back	31.7 (113)	32.9 (34)	16.4 (11)	41.3 (37)	41.8 (31)	
Groin	16.0 (57)	19.3 (21)	24.5 (14)	12.2 (12)	12.3 (10)	
Abdomen	9.0 (32)	10.3 (11)	4.3 (3)	10.4 (10)	9.2 (8)	
Chest	7.0 (25)	10.7 (12)	5.8 (3)	2.9 (3)	8.3 (7)	
Present now (yes)	52.1 (182)	61.2 (67)	49.6 (30)	46.9 (45)	45.0 (40)	0.16
Timing of onset						
<1 month	16.4 (55)	37.7 (28)	11.9 (9)	10.9 (10)	7.4 (8)	0.0003
1–12 months	23.6 (90)	33.7 (40)	22.2 (13)	15.1 (17)	19.4 (20)	
>12 months	60.0 (211)	38.6 (44)	65.9 (39)	73.9 (72)	73.2 (56)	
Type of condition						
Masses	23.8 (80)	31.7 (34)	15.9 (10)	17.1 (18)	26.1 (18)	0.057
Wound (injury)	19.7 (72)	10.1 (12)	18.5 (11)	27.2 (27)	26.7 (22)	
Acquired deformity	16.6 (66)	11.4 (15)	(14)	17.9 (18)	(19)	
Wound (non-injury)	14.8 (54)	15.1 (17)	26.5 (15)	13.2 (14)	8.2 (8)	
Burn	12.5 (42)	16.3 (17)	6.8 (4)	12.9 (12)	9.6 (9)	
Congenital deformity	7.6 (24)	10.4 (11)	8.3 (4)	5.5 (4)	5.3 (5)	
Abdominal problems	4.7 (18)	4.9 (6)	4.5 (3)	6.2 (6)	2.3 (3)	
Untreated surgical condition (yes)	48.9 (174)	55.4 (61)	54.4 (33)	49.5 (48)	36.5 (32)	0.11
*Health care sought and care received*						
Healthcare sought						
Yes	74.2 (264)	75.0 (84)	78.7 (48)	64.6 (64)	81.0 (68)	0.060
No	25.8 (92)	25.0 (28)	21.3 (13)	35.4 (35)	19.0 (16)	
Traditional healer sought						
Yes	19.7 (69)	15.7 (17)	20.0 (12)	23.5 (23)	20.2 (17)	0.58
No	80.3 (281)	84.3 (91)	80.0 (48)	76.5 (75)	79.8 (67)	
Type of care received^a^						
No care	17.0 (45)	23.8 (20)	20.8 (10)	10.9 (7)	11.8 (8)	–
Major procedure	7.6 (20)	3.6 (3)	10.4 (5)	7.8 (5)	10.3 (7)	
Minor procedure	71.2 (188)	66.7 (56)	62.5 (30)	81.3 (52)	73.5 (50)	
Referred	4.2 (11)	6.0 (5)	6.3 (3)	0.0 (0)	4.4 (3)	
Reason for no care^b^						
No money	57.1 (32)	60.0 (15)	76.9 (10)	42.9 (3)	36.4 (4)	–
No perceived need	25.0 (14)	24.0 (6)	7.7 (1)	42.9 (3)	36.4 (4)	
No facility/personnel/equipment	16.1 (9)	20.0 (5)	15.4 (2)	14.3 (1)	9.1 (1)	
No transport	14.3 (8)	12.0 (3)	30.8 (4)	0.0 (0)	9.1 (1)	
Lack of social support	8.9 (5)	12.0 (3)	7.7 (1)	0.0 (0)	9.1 (1)	
Fear	3.6 (2)	4.0 (1)	0.0 (0)	0.0 (0)	9.1 (1)	
No time	1.8 (1)	0.0 (0)	0.0 (0)	0.0 (0)	9.1 (1)	
Reason for not seeking care^c^						
No money	51.1 (47)	39.3 (11)	69.2 (9)	62.9 (22)	31.3 (5)	–
No perceived need	28.3 (26)	35.7 (10)	30.8 (4)	14.3 (5)	43.8 (7)	
Fear	20.7 (19)	14.3 (4)	23.1 (3)	17.1 (6)	37.5 (6)	
No transport	18.5 (17)	17.9 (5)	15.4 (2)	25.7 (9)	6.3 (1)	
Lack of social support	16.3 (15)	17.9 (5)	15.4 (2)	20.0 (7)	6.3 (1)	
No time	9.8 (9)	17.9 (5)	0.0 (0)	8.6 (3)	6.3 (1)	
No facility/personnel/equipment	4.3 (4)	0.0 (0)	15.4 (2)	5.7 (2)	0.0 (0)	

39 children reported 2 conditions, 4 children reported 3 conditions, and 1 child reported 5 conditions

^a^Among those who sought healthcare (*n* = 264)

^b^Among those who sought healthcare but did not receive care (*n* = 56). Multiple responses were allowed per condition. Denominator of percentage is number of conditions

^c^Among those who did not seek healthcare (*n* = 92). Multiple responses were allowed per condition. Denominator of percentage is the number of conditions

The majority of children sought formal healthcare (74.2 %, 264/356), and only 19.7 % sought care from a traditional healer (69/356). Of those who sought formal care, 71.2 % underwent a minor procedure and 17.0 % received no care. Of those who sought care, but did not receive any, the primary reasons were lack of money (57.1 %), no perceived need for surgical care (25.0 %), and lack of available personnel and equipment (16.1 %). Of those who did not seek care, the primary reasons included lack of money (51.1 %), no perceived need (28.3 %), and fear (20.7 %).

### Characteristics of children with surgical conditions by treatment status (*n* = 305)

Of the 356 surgical conditions, 174 (48.9 %) were untreated (Table 3). The majority of abdomen and chest conditions were untreated (71.9 and 72.0 %, respectively). Nearly, all congenital deformities remained untreated (91.7 %), and masses and abdominal problems were less likely to be treated compared with burns and wounds (*p* < 0.0001). Those with untreated conditions had higher rates of traditional healer use (25.9 versus 13.2 %, *p* = 0.002) and lower rates of seeking formal health care (54.6 versus 92.9 %, *p* < 0.0001). Children with untreated conditions who did seek health care cited lack of money as the primary reason for not receiving care.

**Table 3:** Clinical characteristics and healthcare seeking for children with surgical conditions, by treatment status

	Total % (*n*)	Treated % (*n*)	Untreated % (*n*)	Proportion untreated %	*p*
Number of individuals	305	48.9 (149)	51.1 (156)		
Number of conditions	356	51.1 (182)	48.9 (174)		
*Characteristics of condition*					
Location					
Face and neck	36.2 (129)	40.7 (74)	31.6 (55)	42.6	0.011
Extremity and back	31.7 (113)	34.1 (62)	29.3 (51)	45.1	
Groin	16.0 (57)	16.5 (30)	15.5 (27)	47.4	
Abdomen	9.0 (32)	4.9 (9)	13.2 (23)	71.9	
Chest	7.0 (25)	3.8 (7)	10.3 (18)	72.0	
Timing of onset					0.15
<1 month	16.4 (55)	14.3 (26)	16.7 (29)	52.7	
1–12 months	23.6 (90)	29.7 (54)	20.7 (36)	40.0	
>12 months	60.0 (211)	56.0 (102)	62.6 (109)	51.7	
Type of condition					
Masses	23.8 (80)	17.6 (32)	27.6 (48)	60.0	<0.0001
Wound (injury)	19.7 (72)	29.7 (54)	10.3 (18)	25.0	
Acquired deformity	16.6 (66)	15.9 (29)	11.2 (37)	56.0	
Wound (non-injury)	14.8 (54)	18.7 (34)	11.5 (20)	37.0	
Burn	12.5 (42)	14.3 (26)	9.2 (16)	38.1	
Congenital deformity	7.6 (24)	1.1 (2)	12.6 (22)	91.7	
Abdominal problems	4.7 (18)	2.7 (5)	7.5 (13)	72.2	
*Health care sought and care received*					
Healthcare sought					
Yes	74.2 (264)	92.9 (169)	54.6 (95)		<0.0001
No	25.8 (92)	7.1 (13)	45.4 (79)		
Traditional healer sought					
Yes	19.7 (69)	13.2 (24)	25.9 (45)		0.002
No	80.3 (281)	85.2 (155)	72.4 (126)		
Type of care received^a^					
None/no surgical care	17.0 (45)	2.4 (4)	43.2 (41)		–
Major procedure	7.6 (20)	8.9 (15)	5.3 (5)		
Minor procedure	71.2 (188)	88.8 (150)	40.0 (38)		
Referred	4.2 (11)	0 (0)	11.6 (11)		
Reason for no care^b^					
No money	57.1 (32)	25.0 (1)	59.6 (31)		–
No perceived need	25.0 (14)	75.0 (3)	21.2 (11)		
No facility/personnel/equipment	16.1 (9)	0.0 (0)	17.3 (9)		
No transport	14.3 (8)	0.0 (0)	15.4 (8)		
Lack of social support	8.9 (5)	0.0 (0)	9.6 (5)		
Fear	3.6 (2)	0.0 (0)	3.8 (2)		
No time	1.8 (1)	0.0 (0)	1.9 (1)		
Reason for not seeking care^c^					
No money	51.1 (47)	38.5 (5)	53.2 (42)		–
No perceived need	28.3 (26)	38.5 (5)	26.6 (21)		
Fear	20.7 (19)	15.4 (2)	21.5 (17)		
No transport	18.5 (17)	15.4 (2)	19.0 (15)		
Lack of social support	16.3 (15)	7.7 (1)	17.7 (14)		
No time	9.8 (9)	0.0 (0)	11.4 (9)		
No facility/personnel/equipment	4.3 (4)	0.0 (0)	5.1 (4)		

^a^Among those who sought healthcare (*n* = 264)

^b^Among those who sought healthcare, but did not receive care (*n* = 56). Multiple responses were allowed per condition. Denominator of percentage is number of conditions

^c^Among those who did not seek healthcare (*n* = 92). Multiple responses were allowed per condition. Denominator of percentage is the number of conditions

### Characteristics of children who died with surgical conditions (*n* = 28)

Of 90 reported pediatric deaths, 31.1 % (28) were due to a surgical condition. The majority of deaths were male (71.4 %) and less than 6 years of age (57.1 %). Stillbirth and acquired deformity were the most common causes of death. Over half of deaths occurred at home, but 92.8 % sought health care prior to death. Of those who sought health care, 69.3 % underwent either a major procedure or minor procedure, but 23.0 % did not receive any care. Lack of money and time were the most frequent reasons for not receiving care.

## Discussion

In this study, we show that 14 % of children in Uganda have had a surgical condition and only half of these children have received treatment for their condition. Similar SOSAS studies in Sierra Leone, Rwanda, and Nepal estimated the unmet pediatric surgical need at 17.6, 6.3, and 6.0 %, respectively, compared with 7.2 % in Uganda [3–5]. We show similar rates of unmet pediatric surgical need compared with other low-income countries. Extrapolating these estimates to the current Ugandan population [17] results in 2.6 million children with a surgical condition at some point in their lifetime and 1.3 million children with a current need for surgical care. The current surgical need far outweighs the current capacity with three practicing pediatric surgeons, resulting in a growing backlog of necessary procedures. Identifying the burden of pediatric surgical disease is only the first step in improving pediatric surgical care. To address the identified burden, strides must be made in surgical infrastructure, addressing social barriers to care, and training the surgical workforce [7, 20].

Surgical conditions requiring specialty surgical care, including masses, congenital deformities, genitourinary conditions, and abdominal conditions, were more likely to remain untreated compared with the conditions requiring less specialized care, such as wound care. In a study completed on the pediatric surgery unit at the national referral hospital in Kampala, Uganda, 53 % of neonates underwent surgery and those that underwent surgery had much lower mortality (13 %) compared with those who did not undergo surgery (55 %). Badrinath et al. speculate that patients who did not undergo surgery were sicker patients and did no undergo surgery due to lack of critical care resources [21]. Even at the national referral hospital, where two of the three pediatric surgeons practice, many children do not receive surgery due to lack of infrastructure. To bridge the gap of unmet pediatric surgical need, steps must be made in diagnostics, critical care, and curative services by specialized providers, rather than just expansion of basic medical care. In a cost-effectiveness analysis, pediatric inguinal hernia repairs performed in Uganda had a cost-effectiveness ratio of $12.41 per DALY averted, compared with $41 for insecticide-treated bed nets to prevent malaria [22]. It is worth investing in surgical infrastructure to address the large burden of pediatric surgical conditions.

Children with untreated surgical conditions cited lack of money as the primary reason for not receiving or seeking care. All services at public health facilities are nominally free of charge; however, there is often a lack of appropriate surgical supplies, such as sutures, implantable mesh, and anesthetic medications [23]. If these consumables are not available, patients’ families are asked to purchase them, creating insurmountable barriers. Of the 11 individuals who were referred to a higher level of care, none of them had their conditions treated. This points to a broken referral system, in which patients and their caregivers are asked to travel a long distance, leaving their social support system, to arrive at a referral hospital that might not even have the available personnel or equipment to treat their condition. Resource-limited countries must invest in essential surgical care [24] to ensure that cost is not a barrier to surgical care.

Interestingly, many children did not receive surgical care due to lack of a perceived need for surgery. Although it is unclear why patients’ families did not feel their child needed surgical care, it is likely that they believed the condition could be resolved by medication alone or would resolve on its own. This points to the necessity of community education on common surgical conditions. In addition, those with untreated conditions were more likely to have sought care from a traditional healer. Through partnerships between village health workers, traditional healers, and surgical providers, communities can improve understanding of surgical conditions and gain confidence in the healthcare system’s ability to provide surgical care to increase healthcare seeking, when needed.

Finally, lack of an adequate pediatric surgical workforce severely limits meeting the unmet burden of disease. In addition to lack of money and lack of perceived need, lack of personnel and equipment was a common cause that children did not receive surgical care. With three practicing pediatric surgeons in Uganda, there are 0.017 per 100,000 children, far below the accepted density of 1 per 100,000 children in North America [25]. Uganda is only able to train one pediatric surgeon per year through COSECSA, and the pediatric fellows must leave the country for a year to complete their training. Through partnerships with North American universities, the general surgical and neurosurgical workforce has vastly expanded over the last 5 years [26, 27]. Similar strides must be made to improve training of pediatric surgeons. Geographic distribution of available surgeons must also be addressed. Children with treated conditions were more likely to live in the Central region. Two of the three pediatric surgeons practice in Kampala and the third practices in the Eastern region. There are no pediatric surgeons in the North, where unmet pediatric surgical need is highest. As more pediatric surgeons and anesthetists are trained, they must be incentivized to practice in areas other than the capital city of Kampala.

The SOSAS survey was designed as a comprehensive tool to provide a population estimate of the burden of surgical disease. There are a few limitations that must be acknowledged. Using the PMA2020 platform provided competent researchers and data collectors, but the majority of them lacked a health professions degree. We mitigated this lack of knowledge using experienced surgeons and medical/surgical trainees, with knowledge of Ugandan surgical disease presentation, to interpret open-ended responses. This likely led to a higher level of specificity in the data given that many conditions were excluded based on their description, but did not change the sensitivity. The survey also likely underestimates surgical need, as many conditions, such as cancer, are asymptomatic until late presentation.

The reliance on self-reporting is a common criticism of the SOSAS method, though recently, the survey was compared with a visual examination that agreed with participant self-reporting in 94.6 % of cases [28]. However, the validation occurred among those who consented to a visual examination, included only certain anatomical regions, excluding groin and chest examinations, and usage of a physical examination rather than a full surgical consultation. These limitations may bias the results, and future studies quantifying surgical burden should attempt to overcome these potential biases regarding diagnostic accuracies.

## Conclusions

Throughout Africa, children comprise 46 % of the population [29], thus highlighting the need for appropriate infrastructure, supplies, and personnel to provide needed surgery, particularly in high burden areas, such as Uganda, where 55 % of surgical needs are attributable to the pediatric population. Our data highlight the need for improved provision of specialty pediatric surgical care combined with public health prevention programs to prevent pediatric trauma. The next steps are to design interventions to improve out-of-pocket cost of surgical care, change perceptions of surgical need, and create a pediatric surgical workforce able to meet the need.
